# Human Social Genomics

**DOI:** 10.1371/journal.pgen.1004601

**Published:** 2014-08-28

**Authors:** Steven W. Cole

**Affiliations:** 1 Department of Medicine, Division of Hematology-Oncology, UCLA School of Medicine, University of California, Los Angeles, Los Angeles, California, United States of America; 2 Norman Cousins Center, University of California, Los Angeles, Los Angeles, California, United States of America; 3 UCLA Molecular Biology Institute, University of California, Los Angeles, Los Angeles, California, United States of America; 4 Jonsson Comprehensive Cancer Center, University of California, Los Angeles, Los Angeles, California, United States of America; 5 UCLA AIDS Institute, University of California, Los Angeles, Los Angeles, California, United States of America; Georgia Institute of Technology, United States of America

## Abstract

A growing literature in human social genomics has begun to analyze how everyday life circumstances influence human gene expression. Social-environmental conditions such as urbanity, low socioeconomic status, social isolation, social threat, and low or unstable social status have been found to associate with differential expression of hundreds of gene transcripts in leukocytes and diseased tissues such as metastatic cancers. In leukocytes, diverse types of social adversity evoke a common conserved transcriptional response to adversity (CTRA) characterized by increased expression of proinflammatory genes and decreased expression of genes involved in innate antiviral responses and antibody synthesis. Mechanistic analyses have mapped the neural “social signal transduction” pathways that stimulate CTRA gene expression in response to social threat and may contribute to social gradients in health. Research has also begun to analyze the functional genomics of optimal health and thriving. Two emerging opportunities now stand to revolutionize our understanding of the everyday life of the human genome: network genomics analyses examining how systems-level capabilities emerge from groups of individual socially sensitive genomes and near-real-time transcriptional biofeedback to empirically optimize individual well-being in the context of the unique genetic, geographic, historical, developmental, and social contexts that jointly shape the transcriptional realization of our innate human genomic potential for thriving.

## Introduction

The spectacular adaptive success of *Homo sapiens* is attributable in large part to our capacity to self-organize into complex social systems or “metaorganisms” [1]–[3]. Research in human social genomics has begun to clarify how these extraorganismic social systems reciprocally regulate our intraorganismic physiologic function by modulating tissue-specific programs of gene expression [3]–[5]. Social regulation of gene expression has long been observed in animal models of morphological plasticity such as worker bee maturation into guards and scouts, cichlid sex switching, and status-dependent changes in body size, coloring, brain development, immune response, and reproductive capacity [6]–[9]. However, scientists, policy makers, and the general public have long wondered how such animal dynamics might pertain to everyday human life. Studies of human social genomics are now clarifying which specific types of human genes are subject to social regulation and mapping the social signal transduction pathways that mediate these effects. The results of these analyses are shedding new light on the molecular basis for social influences on individual heath, the genomic basis for human thriving, and the metagenomic capabilities that emerge from networked communities of socially sensitive genomes and underpin human group selection and the evolution of our hypersocial life history strategy [2], [10].

## Human Social Genomics

**Figure 1:**
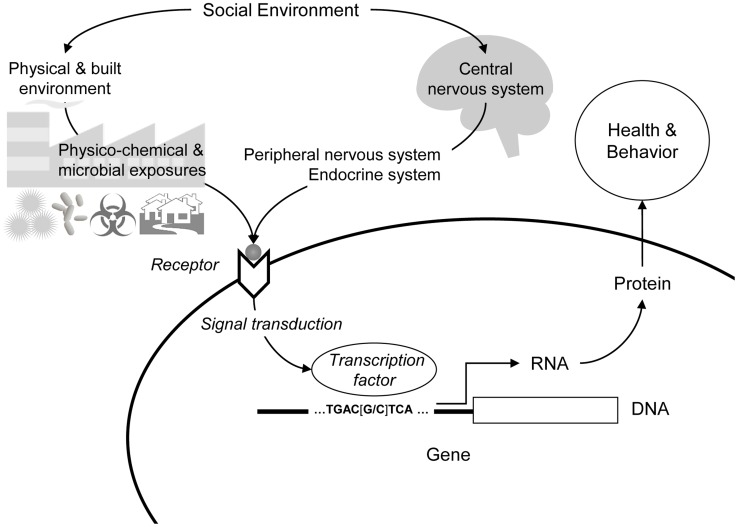
Social regulation of human gene expression. Social environments can influence human gene expression via physicochemical processes (e.g., toxins, pollutants, and microbes) and psychological processes (e.g., experiences of threat or uncertainty) that trigger neural and endocrine responses (e.g., activation of the sympathetic nervous system). In both cases, biochemical mediators engage cellular receptor systems, which activate intracellular signal transduction pathways culminating in the activation (or repression) of transcription factors that proximally regulate the transcription of genes bearing response elements for that particular factor. The gene regulatory “wiring diagram” that maps specific biochemical signals to specific gene expression responses represents an evolved genomic program that was presumably adaptive under ancestral conditions but may have distinct maladaptive effects in the very different social environments of contemporary human life.

Initial indications that social environments might significantly affect the functional activity of the human genome came from studies dissecting leukocyte gene expression profiles into genetic and environmental components [4], [11]. Gibson and colleagues found that ∼5% of genes expressed in leukocytes showed appreciable genetic regulation (e.g., via expression quantitative trait loci), whereas >50% showed significant differences in expression across pastoral, rural, and urban social environments [11]. These results documented a substantial relationship between general social context and genome function, and motivated further analysis of the specific features of the social environment that drive the observed differences in gene expression (e.g., physicochemical stimuli, microbial exposures, and social/psychological influences on physiology). Parallel studies on the transcriptional correlates of social disparities in health subsequently suggested that both physical and psychological processes contribute to the net effect of a given social environment, with each mechanism activating some distinct gene modules as well as a conserved generalized response to adverse life circumstances (Figure 1) [3], [5], [12].

A prototypic example comes from studies of social isolation, which is one of the most robust epidemiologic risk factors for chronic illness and mortality [13]. Genome-wide transcriptional profiling of leukocytes from people experiencing chronic social isolation identified >200 genes that showed >50% difference in average expression levels relative to those observed in socially integrated people [14], [15]. Genes up-regulated in socially isolated individuals included a set of transcripts that play a central role in inflammation (e.g., *IL1B*, *IL8*, *PTGS2*), whereas down-regulated transcripts were involved in Type I interferon innate antiviral responses (e.g., *ISG*, *IFI*, *MX*, and *OAS* family genes) and in antibody production (e.g., *IGL*, *IGH*, *IGJ*, and *IGK*) [5], [14], [15]. Epidemiologists have long debated whether the health effects of social isolation stem predominately from a lack of social contact per se (i.e., reduced network size, economic opportunity, personal assistance, and interpersonal contact) or from the subjective experience of being lonely and disconnected from the rest of society and the threat/stress reactions that ensue [1], [13], [16]. These transcriptome analyses suggested that both subjective and objective isolation likely play a role but do so through distinct gene regulatory pathways. Objective isolation was associated with reduced expression of antibody synthesis genes (perhaps due to reduced exposure to socially transmitted microbes), whereas subjective isolation associated with increased expression of proinflammatory genes and reduced expression of Type I interferon genes and transcripts specifically involved in synthesis of immunoglobulin G_1_ (IgG_1_) antibodies (a pattern subsequently linked to fight-or-flight threat responses from the sympathetic nervous system) [3], [5].

## A Conserved Transcriptional Response to Adversity

Following the initial analyses of social isolation, a diverse array of studies has begun to document similar leukocyte transcriptome shifts in other adverse social conditions, including low socioeconomic status (SES) [9], [17]–[20], chronic stress (e.g., care-giving for a dying spouse) [21], [22], bereavement [23], post-traumatic stress disorder (PTSD) [24], [25], and cancer diagnosis [26], [27]. Across these diverse forms of adversity, a common pattern of conserved transcriptional response to adversity (CTRA) has emerged, including increased expression of proinflammatory genes and decreased expression of genes involved in Type I interferon innate antiviral responses and IgG antibody synthesis [3], [5], [28].

Subsequent studies using experimental animal models has shown that CTRA gene expression profiles can be induced in leukocytes by repeated social threat [9], unstable social hierarchies [8], [29], and low social status [7]. Randomized controlled studies in humans have also shown that CTRA gene expression profiles can be suppressed by interventions such as cognitive behavioral stress management [26], meditation [30], [31], yoga [32], and Tai Chi [33].

**Figure 2:**
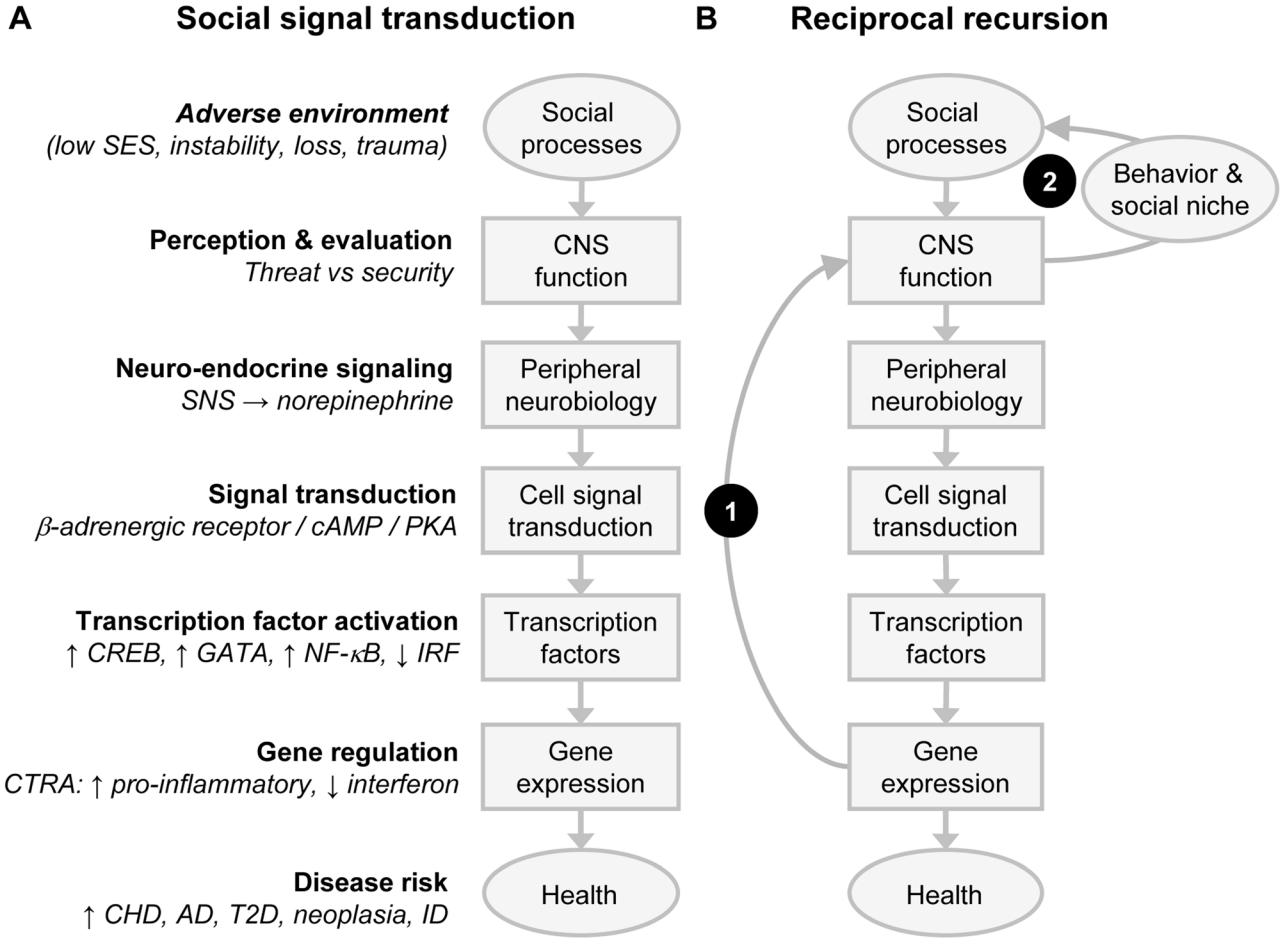
Social signal transduction and recursive network genomics. (A) A simple (acyclic) social signal transduction pathway maps adverse social conditions onto activation of the conserved transcriptional response to adversity (CTRA) in leukocytes. Brain-mediated perceptions of social threat activate the sympathetic nervous system (SNS), leading to release of norepinephrine (NE) at SNS nerve terminals, activation of β-adrenergic receptors on adjacent cells, and stimulation or repression of specific transcription factors in response to the cyclic 3′-5′ adenosine monophosphate/protein kinase A (cAMP/PKA) signaling pathway. β-adrenergic-responsive transcription factors induce the CTRA gene expression program by stimulating transcription of genes encoding proinflammatory cytokines and suppressing transcription of genes encoding Type I interferons and IgG antibodies. CTRA gene expression programs prepare the body to respond to wounding injury and bacterial infections but may promote chronic illnesses such as cardiovascular disease (CVD), Alzheimer disease (AD), Type II diabetes (T2D), and metastatic cancer while undermining host resistance to virally mediated infectious diseases (ID). (B) Superimposed effects of reciprocal endogenous and exogenous recursive feedback on the social signal transduction pathway can propagate the impact of a transient adverse environmental shock. In this system, a transient environmental threat activates the core social signal transduction pathway to stimulate transcription of proinflammatory cytokine genes, as part of the CTRA. Arc 1 shows an endogenous biological feedback loop in which the proinflammatory gene products signal the brain to activate a programmed set of sickness behaviors that include reduced social motivation, fatigue, anhedonia, and negative emotional states. Arc 2 shows how the resulting reductions in individual social behavior and altered social niche selection evoke less supportive and more hostile responses from the surrounding social network and thereby create a more adverse social environment. Effects of the exogenous social recursion loop (Arc 2) are propagated via the core social signal transduction cascade into continued CTRA activation and continued endogenous biological recursion (Arc 1). Reciprocating feedback may thus maintain the system in a new dynamic equilibrium that maintains altered endogenous inflammation and exogenous social influence long after the initiating transient shock has passed. Similar recursive feedback can occur at every level of the social signal transduction cascade, resulting in complex systems dynamics that can trigger persistent sequelae such as PTSD and biological embedding of early life social conditions without requiring any durable genomic modification (e.g., mutation or epigenetic marking). Abbreviations: CHD, coronary heart disease; CNS (central nervous system); IRF, interferon regulatory factor.

Mechanistic studies in animal and cell culture systems have also shown that activation of fight-or-flight signaling pathways in the sympathetic nervous system (SNS) plays a major role in evoking CTRA gene expression profiles (Figure 2A). SNS activation of the CTRA is mediated in large part by β-adrenergic receptors, which stimulate transcription factors such as nuclear factor kappa-light-chain-enhancer of activated B cells (NF-κB), GATA, and cAMP response element-binding protein (CREB) to selectively up-regulate transcription of proinflammatory genes (e.g., *IL6* [19]) while simultaneously inhibiting the activity of transcription factors, such as the interferon response factor family, that control transcription of Type I interferon genes (e.g., *IFNB* [34]). This pleiomorphic modulation of multiple transcription control pathways allows for rapid and relatively focal changes in leukocyte transcriptomes during extended periods of organismic threat and SNS fight-or-flight responses.

A second pathway inducing the CTRA involves broader and more durable changes in the transcriptional underpinnings of immune cell growth and development. Bioinformatic decomposition of transcriptome shifts in the heterogenous leukocyte population initially identified the primary cellular mediators of CTRA transcriptome shifts as myeloid lineage immune cells such as monocytes and dendritic cells [15]. Subsequent analysis of physically isolated leukocyte subpopulations confirmed that monocytes mediate many of the transcriptional effects of human social adversity [21], [25], and mechanistic analyses in animal models determined that the SNS can up-regulate the production of a distinct subpopulation of immature, proinflammatory monocytes by altering hematopoietic processes in the bone marrow [9]. These effects are again mediated by β-adrenergic signaling, which up-regulates transcription of the myelopoietic growth factor granulocyte-macrophage colony-stimulating factor (GM-CSF) (*Csf3*) and thereby enhances monocyte differentiation and development [9]. The resulting proinflammatory skew in the composition of the whole body monocyte pool persists for the life of the cells, which can sequester in reservoirs such as the spleen and reemerge months later in response to social threat [35].

A third pathway by which adverse social conditions can induce persistent transcriptional alterations in immune cells involves up-regulation of the *NGF* gene that supports the growth and differentiation of the SNS nerve fibers innervating lymph nodes [29]. The resulting increase in neurotransmitter delivery to the lymph node increases throughput from the brain to the immune system and thereby induces a persistent shift in the gene regulatory program of the tissue-resident pool of cells (e.g., down-regulating Type I interferon transcription and host resistance to viral infection [29]). In combination with SNS/β-adrenergic regulation of focal transcriptional programs in existing monocytes and de novo monocyte production through myelopoiesis, social threat–induced neoinnervation of lymphoid tissues provides three distinct gene regulatory dynamics that converge to up-regulated CTRA gene expression in immune cells during extended periods of social threat or adversity [36].

There has been great interest in the possibility that epigenetic marks such as histone acetylation or DNA methylation might mediate social influences on human gene expression. Environmental conditions clearly influence epigenetic profiles in human immune cells [37], and some studies have begun to link immune cell DNA methylation profiles to specific social environmental conditions such as low social status [7] and early life stress exposure [38]–[41]. However, those epigenetic profiles are only weakly correlated with differences in immune cell gene expression [7], [41], and much remains to be learned about what role epigenetic dynamics play in social genomic effects such as the CTRA.

Beyond the immune system, studies have recently begun to map relationships between social conditions and gene expression in diseased tissues such as breast, ovarian, and prostate cancers [42]. In these solid epithelial tumors, low social support is associated with increased expression of many gene modules that promote cancer progression and metastasis, including transcripts involved in inflammation, wound repair (epithelial-mesenchymal transition), blood vessel growth (angiogenesis), and resistance to programmed cell death (anoikis and chemotherapy resistance) [42]. Parallel effects have been observed in experimental animal models of cancer, and those analyses again identify a prominent role for SNS activation of β-adrenergic signaling [43], [44]. The specific genes regulated by β-adrenergic signaling in tumor tissues differ in tissue-specific ways from those engaged by β-adrenergic signaling in leukocytes, but they share some common teleological principles in their distinct cellular contexts. In both, chronic threat and SNS signaling activate tissue-specific transcriptional defense programs that enhance organismic mobility and prime wound healing and antimicrobial responses to tissue injury [3], [5], [36]. Some SNS-induced transcriptional responses play central roles in both the systemic leukocyte pool and the localized context of a growing tumor. In the context of metastatic breast cancer, for example, stress effects on tumor metastasis are mediated by β-adrenergic up-regulation of monocytes and macrophages within the tumor microenvironment [44] (i.e., the same dynamic that drives the CTRA transcriptome shift in circulating blood [9]). Other neuroendocrine signaling pathways such as glucocorticoid release from the hypothalamic-pituitary-adrenal system may also play a role in mediating relationships between social conditions and cancer biology [45]. For example, socially isolated animals show glucocorticoid-related increases in breast cancer burden [46], [47], and molecular analyses find glucocorticoid signaling to promote several cancer-related processes including mammary adipocyte metabolism [47], [48], inhibition of tumor suppressor genes such as *TP53* [49], and resistance to chemotherapy-mediated programmed cell death [50], [51]. Interestingly, a key gene involved in mediating these effects shows evidence of recent adaptive variation across human populations [52].

## Genomics of Human Thriving

Transcriptional defense programs such as the CTRA presumably evolved to help adapt molecular physiology to the types of sporadic and transient threats that generally characterized our ancestral environments. In the contemporary human social ecology, however, chronic activation of these transcriptional defense programs by purely symbolic or anticipated threats likely acts to undermine health by promoting inflammation-related chronic diseases such as Type II diabetes, atherosclerosis, neurodegeneration, and metastatic cancer while simultaneously undermining host resistance to viral infections [3], [5], [19], [36]. However, genomes evolve to help us thrive and proliferate, not to generate disease [53]. As such, mapping the evolved regulatory logic of the human genome should have more to tell us about human well-being and the biology of thriving than it does about disease per se. If we seek to avoid the chronic activation of costly molecular defense programs such as the CTRA, what is the best way for us to lead our lives?

One recent study by Fredrickson and colleagues took this approach to ask which kind of “happiness” might best oppose the CTRA [54]. This was a more complex question than it might seem because several different forms of human happiness exist, and they imply very different approaches to promoting human health. Philosophers have long distinguished between a hedonic form of well-being generated by the pursuit of positive emotional experiences and consummatory self-gratification (i.e., self-focused happiness) and a more eudaimonic form of well-being that stems from devoting one's efforts to a noble cause or purpose outside the self (i.e., self-transcendent happiness). Fredrickson and colleagues found that high levels of eudaimonic well-being were associated with significantly lower levels of CTRA-related gene expression (i.e., a more favorable, or less threatened, molecular profile) [54]. In contrast, people who showed comparatively high levels of hedonic well-being relative to their level of eudaimonic well-being showed significantly elevated CTRA gene expression (above and beyond any effects of hedonistic behavioral factors such as smoking, alcohol consumption, or adiposity). Many psychological theories propose that high levels of life satisfaction and positive affect and low levels of experienced stress (i.e., hedonic well-being) should promote physical health. This genomics-based analysis implies that positive affect alone may not be sufficient and that interventions targeting the self-transcendent approach to psychological well-being may provide the greatest parallel increments to molecular well-being.

What else might molecular indicators of well-being tell us about the best way for humans to live? Several randomized controlled experimental studies have shown that contemplative practices such as mindfulness meditation, physical practices such as yoga and Tai Chi, and cognitive-behavioral stress management programs can all reduce proinflammatory gene expression in people confronting significant life adversity [26], [30]–[33]. Analyses comparing long-term meditators to novice controls have also shown such differences [55]–[58] and imply that even under favorable life circumstances there may exist practical opportunities to enhance molecular well-being. Transcriptomic measures of human well-being are just beginning to enter behavioral science, but their use should spread rapidly because they yield information that is qualitatively distinct from that available through other well-being assessments such as conscious introspection. Fredrickson et al., for example, found striking differences in the gene transcriptional correlates of eudaimonic and hedonic happiness despite the fact that these two types of happiness did not differ at all in self-reported measures of emotional well-being.

## Network Genomics and Social Recursion

Although studies have begun to examine both the positive and negative impacts of broad social climates such as SES and social stability, little is presently known about how these influences are transmitted via concrete social networks. It should be possible to map the flow of social genomic influences through networks of individuals and observe the contagious development of coordinated “cultures” of gene expression. Network genomics analyses could clarify a wide range of issues ranging from the distributed nature of individual resilience to basic questions regarding human evolution and gene-culture interaction. Emergent systems-level capacities lie at the heart of individual well-being, group selection, and our hypersocial life history strategy [2], [10]. Network genomics provides a concrete framework for analyzing how individual socially sensitive human genomes spontaneously self-assemble into coherent metagenomic networks with new systems-level capabilities.

Network genomics analyses are complicated by the fact that people actively shape and select their social environments. These active network dynamics are mediated by brains and nervous systems that are themselves transcriptionally plastic and thus sensitive to both biological and social influences [59], [60]. Figure 2B shows how this kind of transcriptional plasticity in the neural underpinnings of social signal transduction can create recursive feedback systems that can persist over time in stable attractor states and can occasionally shift rapidly to new stable equilibrium in response to a transient environmental shock such as a traumatic event. Figure 2A depicts the simple acyclic social signal transduction pathway that translates social threat into CTRA gene expression. Figure 2B superimposes two empirically documented positive feedback loops—one endogenous biological loop in which proinflammatory gene products from the CTRA signal back to the brain to reduce prosocial motivation, perception, and behavior (Arc 1) [61]–[63] and a second exogenous social loop (Arc 2) in which altered behaviors evoke more suspicious, distant, and hostile responses from the surrounding social environment [1]. Adverse social responses propagate the individual's experience of social threat and thus promote continued CTRA signaling, continued proinflammatory feedback to the brain, continued suppression of prosocial behavior, continued adverse social reactions, and so forth. Absent either one of these feedback loops, the effects of a transient environmental shock would generally damp and decay over time. When both loops are present, however, the output of one becomes an input to the other, and they can reciprocally propagate defensive gene expression responses long after the precipitating shock has passed. This provides a purely functional mechanism by which transient environmental conditions can produce persistent biological, psychological, and social sequelae (e.g., as in PTSD or biological embedding of adverse early life social conditions) without involving any persistent DNA modification (e.g., epigenetic marking).

## Prescriptive Genomics

Functional genomics occupies a unique nexus between basic human nature, as reflected in the evolved regulatory programming of our genome to generate successful human life, and the environmentally contingent realization of that innate potential for thriving [53]. As we learn more about how everyday life circumstances influence the transcriptional realization of our genomic potential, human social genomics comes to acquire a certain prescriptive aspect. Disease, death, threat, resilience, thriving, and generativity are not value-neutral in evolutionary terms nor are they in humanitarian terms. As such, molecular indicators of human well-being such as the CTRA and blood-informative transcripts (BIT) [12], [64] may add a new dimension to social, cultural, and political discourse by providing objective leading indicators of human biological well-being that can help gauge the extent to which particular social conditions are congruent with basic human nature [10] and help realize our genomically endowed potential for thriving.

Perhaps the most transformative implication of prescriptive social genomics lies in the new opportunities it provides to us as individuals as we seek to optimize our own personal well-being. Given the diverse array of genetic, developmental, historical, and socioenvironmental factors that shape our realized transcriptomes, there may not exist any single intervention that enhances biological well-being for everyone. However, we should still be able to empirically optimize our personal well-being by selecting the lifestyle components that best work for us in the context of our own particular life circumstances. Determining what works at the individual level has historically been complicated by the fact that the primary objective indicators of well-being involve disease and mortality events that generally occur decades after their environmental precipitants and leave little room for a do-over. Our emerging ability to monitor the molecular underpinnings of human well-being in everyday life using portable real-time RNA sequencing interpreted through evaluated molecular biomarkers such as the CTRA and BIT gene sets provides a wholly new opportunity to discover what works for us personally, long before those molecular dynamics coalesce into overt disease. Continuous real-time molecular biofeedback opens up a new era of human molecular self-awareness and biological self-determination. If we judge societies at least in part by the extent to which they help each of us realize our genomically endowed potential for well-being, then the molecularly quantified self could represent one of our own society's most significant achievements. After all, each of our human genomes is fundamentally a system for converting environmental information into molecular resources according to the accumulated wisdom of 4 million years of hominid evolution, and those who stand to gain most from the insights they afford are we whose lives they create.
